# TFBSFootprinter: a multiomics tool for prediction of transcription factor binding sites in vertebrate species

**DOI:** 10.1080/21541264.2025.2521764

**Published:** 2025-07-11

**Authors:** Harlan R. Barker, Seppo Parkkila, MarttiE.E. Tolvanen

**Affiliations:** a Tampere University Hospital and Faculty of Medicine and Health Technology, Tampere University, Tampere, Finland; b Department of Clinical Chemistry, Fimlab Laboratories PLC, Tampere University Hospital, Tampere, Finland; c Disease Networks Unit, Faculty of Biochemistry and Molecular Medicine, University of Oulu, Oulu, Finland; d Department of Computing, University of Turku, Turku, Finland

**Keywords:** Transcription factor binding site, promoter, gene regulation, multiomics, vertebrate, bioinformatics

## Abstract

**Background:**

Transcription factor (TF) proteins play a critical role in the regulation of eukaryotic gene expression via sequence-specific binding to genomic locations known as transcription factor binding sites (TFBSs). Accurate prediction of TFBSs is essential for understanding gene regulation, disease mechanisms, and drug discovery. These studies are therefore relevant not only in humans but also in model organisms and domesticated and wild animals. However, current tools for the automatic analysis of TFBSs in gene promoter regions are limited in their usability across multiple species. To our knowledge, no tools currently exist that allow for automatic analysis of TFBSs in gene promoter regions for many species.

**Methodology and Findings:**

The TFBSFootprinter tool combines multiomic transcription-relevant data for more accurate prediction of functional TFBSs in 317 vertebrate species. In humans, this includes vertebrate sequence conservation (GERP), proximity to transcription start sites (FANTOM5), correlation of expression between target genes and TFs predicted to bind promoters (FANTOM5), overlap with ChIP-Seq TF metaclusters (GTRD), overlap with ATAC-Seq peaks (ENCODE), eQTLs (GTEx), and the observed/expected CpG ratio (Ensembl). In non-human vertebrates, this includes GERP, proximity to transcription start sites, and CpG ratio.

TFBSFootprinter analyses are based on the Ensembl transcript ID for simplicity of use and require minimal setup steps. Benchmarking of the TFBSFootprinter on a manually curated and experimentally verified dataset of TFBSs produced superior results when using all multiomic data (average area under the receiver operating characteristic curve, 0.881), compared with DeepBind (0.798), DeepSEA (0.682), FIMO (0.817) and traditional PWM (0.854). The results were further improved by selecting the best overall combination of multiomic data (0.910). Additionally, we determined combinations of multiomic data that provide the best model of binding for each TF. TFBSFootprinter is available as Conda and Python packages.

## Introduction

Transcription factor (TF) proteins play a critical role in the regulation of eukaryotic gene expression by sequence-specific binding to short stretches of DNA (6–24 bp) known as transcription factor binding sites (TFBSs) [1] which can comprise larger genomic locations known as cis-regulatory elements (CREs) [2]. Promoters and enhancers are the most common types of CREs and TF binding in these regions is ultimately responsible for activating, enhancing, and repressing gene expression programs [1,2]. Because of the role these proteins play in transcription, the discovery of TFBSs greatly furthers the understanding of many, if not all, biological processes [1]. In previous works we have used TFBS prediction to derive insights about gene expression in studies of wound healing [3], brain tumors [4], and SARS-CoV-2 [5,6].

Many tools have been created to identify TFBSs. Depending on the approach, the extent of incorporation of relevant experimental data varies widely. Early on, the position weight matrix (PWM) was used to represent and predict the binding of proteins to DNA. The PWM can then be used to obtain a likelihood score for a target DNA region, which thus represents the likelihood of a TF binding to that DNA sequence.

In the search for increased accuracy, newer models have improved TFBS prediction by incorporating other relevant biological data, such as 3D structure of DNA [7–11], chromatin accessibility/DNase hypersensitivity sites [12–14], overlap in gene ontology [15], amino acid physicochemical properties [16], and gene expression and chromatin accessibility [17,18]. These alternative models often match or outperform strictly sequence-based models [11,19] in the prediction of TFBSs, although they may involve inefficient and/or underdeveloped technologies compared with the more widely used ChIP-Seq and SELEX approaches. However, this varies by TF, and therefore, it may make sense to derive individual models composed of the most relevant contextual data for each [20]. In addition, algorithms for ab initio motif discovery and enrichment from in vivo data, such as HOMER [21], STEME [22], ProSampler [23], and STREME [24], which are reviewed here [25], are also under development. Correspondingly, several databases catalog TF motifs, most prominently JASPAR [26,27] and TRANSFAC [28].

### TFBSFootprinter incorporates multiomic transcription-relevant data

We sought to identify multiple sources of experimental data relevant to gene expression and TF binding and to incorporate them into a comprehensive model to improve the prediction of functional TFBSs. Specifically, clustering of TFBSs has been shown to be an indicator of functionality [29–31]; conservation of genetic sequences across genomes of related species is one of the most successfully used attributes in the identification of TFBSs [31,32]; proximity to the transcription start site (TSS) is strongly linked to TFBS functionality [33]; correlation of expression between a TF and another gene is an indication of a functional relationship [17,34,35]; variants in noncoding regions have a demonstrated effect on gene expression [36–38]; and variants affecting gene expression are enriched in TFBSs [39]; open chromatin regions (ascertained by ATAC-Seq or DNase sensitivity) correlate with TF binding [40]; and finally, as previously mentioned, significant effort has gone into identifying the actual composition of the binding sites themselves through the use of sequencing of TFBSs (e.g., ChIP-Seq and HT-SELEX) [41,42] as cataloged in several extensive databases, such as ReMap [43], the Cistrome Data Browser [44], and the Gene Transcription Regulation Database (GTRD) [45].

### Ensembl identifier-oriented system of analyses allows analyses in many species

For our tool, the Ensembl transcript ID was chosen as the basic unit of reference. As a result, the tool we present here – TFBSFootprinter – can offer predictions in 317 vertebrates at the time of writing, including many model organisms and domesticated and wild animals (Table 1). From human and model organisms such as mouse and zebrafish to African bush elephant, the catalog can increase as the Ensembl database itself expands. Additionally, it allows the inclusion of important datasets that are gene-centric, such as FANTOM [46] TSSs and expression data, GTEx [36] expression quantitative trait loci (eQTLs), and all annotations that are compiled within Ensembl itself. Finally, the Ensembl transcript ID provides an easy point of reference for a greater audience of scientists, thus increasing the accessibility and utility of the tool.

**Table 1.** Ensembl species in which TFBSFootprinter analysis can be performed.

species name	species common name	species taxon	species assembly
Acanthochromis polyacanthus	spiny chromis	80966	ASM210954v1
Accipiter nisus	Eurasian sparrowhawk	211598	Accipiter nisus ver1.0
Ailuropoda melanoleuca	giant panda	9646	ASM200744v2
Amazona collaria	yellow-billed parrot	241587	ASM394721v1
Amphilophus citrinellus	Midas cichlid	61819	Midas v5
Amphiprion ocellaris	clown anemonefish	80972	ASM2253959v1
Amphiprion percula	orange clownfish	161767	Nemo v1
Anabas testudineus	climbing perch	64144	fAnaTes1.3
Anas platyrhynchos	mallard	8839	ASM874695v1
Anas platyrhynchos platyrhynchos	common mallard	8840	CAU duck1.0
Anas zonorhyncha	Eastern spot-billed duck	75864	ASM222487v1
Anolis carolinensis	green anole	28377	AnoCar2.0v2
Anser brachyrhynchus	pink-footed goose	132585	ASM259213v1
Anser cygnoides	swan goose	8845	GooseV1.0
Aotus nancymaae	Ma’s night monkey	37293	Anan 2.0
Apteryx haastii	Great spotted kiwi	8823	aptHaa1
Apteryx owenii	little spotted kiwi	8824	aptOwe1
Apteryx rowi	Okarito brown kiwi	308060	aptRow1
Aquila chrysaetos chrysaetos	golden eagle	223781	bAquChr1.2
Astatotilapia calliptera	eastern happy	8154	fAstCal1.3
Astyanax mexicanus	Mexican tetra	7994	Astyanax mexicanus-2.0
Astyanax mexicanus pachon	Pachon cavefish	7994	Astyanax mexicanus-1.0.2
Athene cunicularia	burrowing owl	194338	athCun1
Balaenoptera musculus	Blue whale	9771	mBalMus1.v2
Betta splendens	Siamese fighting fish	158456	fBetSpl5.2
Bison bison bison	American bison	43346	Bison UMD1.0
Bos grunniens	domestic yak	30521	LU Bosgru v3.0
Bos indicus hybrid	hybrid cattle	30522	UOA Brahman 1
Bos mutus	wild yak	72004	BosGru v2.0
Bos taurus	cattle	9913	ARS-UCD1.3
Bos taurus hybrid	hybrid cattle	30522	UOA Angus 1
Bubo bubo	Eurasian eagle-owl	30461	BubBub1.0
Buteo japonicus	eastern buzzard	224669	ButJap1.0
Caenorhabditis elegans	C.elegans	6239	WBcel235
Cairina moschata domestica	muscovy Duck (domestic type)	1240228	CaiMos1.0
Calidris pugnax	ruff	198806	ASM143184v1
Calidris pygmaea	Spoon-billed sandpiper	425635	ASM369795v1
Callithrix jacchus	white-tufted-ear marmoset	9483	mCalJac1.pat.X
Callorhinchus milii	elephant shark	7868	Callorhinchus milii-6.1.3
Camarhynchus parvulus	small tree finch	87175	Camarhynchus parvulus V1.1
Camelus dromedarius	Arabian camel	9838	CamDro2
Canis lupus dingo	dingo	286419	ASM325472v1
Canis lupus familiaris	dog	9615	ROS Cfam 1.0
Canis lupus familiarisbasenji	dog	9615	Basenji breed-1.1
Canis lupus familiarisboxer	dog	9615	Dog10K Boxer Tasha
Canis lupus familiarisgreatdane	dog	9615	UMICH Zoey 3.1
Canis lupus familiarisgsd	dog	9615	UU Cfam GSD 1.0
Capra hircus	Goat	9925	ARS1
Capra hircus blackbengal	Goat	9925	CVASU BBG 1.0
Carassius auratus	goldfish	7957	ASM336829v1
Carlito syrichta	Philippine tarsier	1868482	Tarsius syrichta-2.0.1
Castor canadensis	American beaver	51338	C.can genome v1.0
Catagonus wagneri	Chacoan peccary	51154	CatWag v2 BIUU UCD
Catharus ustulatus	Swainson’s thrush	91951	bCatUst1.pri
Cavia aperea	Brazilian guinea pig	37548	CavAp1.0
Cavia porcellus	domestic guinea pig	10141	Cavpor3.0
Cebus imitator	Panamanian white-faced capuchin	2715852	Cebus imitator-1.0
Cercocebus atys	Sooty mangabey	9531	Caty 1.0
Cervus hanglu yarkandensis	Yarkand deer	84702	CEY v1
Chelonoidis abingdonii	Abingdon island giant tortoise	106734	ASM359739v1
Chelydra serpentina	Common snapping turtle	8475	Chelydra serpentina-1.0
Chinchilla lanigera	Long-tailed chinchilla	34839	ChiLan1.0
Chlorocebus sabaeus	African green monkey	60711	ChlSab1.1
Choloepus hoffmanni	Hoffmann’s two-fingered sloth	9358	choHof1
Chrysemys picta bellii	Western painted turtle	8478	Chrysemys picta bellii-3.0.3
Chrysolophus pictus	golden pheasant	9089	Chrysolophus pictus GenomeV1.0
Ciona intestinalis	Sea squirt Ciona intestinalis	7719	KH
Ciona savignyi	Sea squirt Ciona savignyi	51511	CSAV2.0
Clupea harengus	Atlantic herring	7950	Ch v2.0.2v2
Colobus angolensis palliatus	Angola colobus	336983	Cang.pa 1.0
Corvus moneduloides	New Caledonian crow	1196302	bCorMon1.pri
Cottoperca gobio	channel bull blenny	56716	fCotGob3.1
Coturnix japonica	Japanese quail	93934	Coturnix japonica 2.0
Cricetulus griseus chok1gshd	Chinese hamster	10029	CHOK1GS HDv1
Cricetulus griseus crigri	Chinese hamster	10029	CriGri 1.0
Cricetulus griseus picr	Chinese hamster	10029	CriGri-PICRH-1.0
Crocodylus porosus	Australian saltwater crocodile	8502	CroPor comp1
Cyanistes caeruleus	blue tit	156563	cyaCae2
Cyclopterus lumpus	lumpfish	8103	fCycLum1.pri
Cynoglossus semilaevis	tongue sole	244447	Cse v1.0
Cyprinodon variegatus	sheepshead minnow	28743	C variegatus-1.0
Cyprinus carpio carpio	common carp	630221	Cypcar WagV4.0
Cyprinus carpio germanmirror	common carp german mirror	7962	German Mirror carp 1.0
Cyprinus carpio hebaored	common carp hebao red	7962	Hebao red carp 1.0
Cyprinus carpio huanghe	common carp huanghe	7962	Hunaghe carp 2.0
Danio rerio	zebrafish	7955	GRCz11
Dasypus novemcinctus	nine-banded armadillo	9361	Dasnov3.0
Delphinapterus leucas	beluga whale	9749	ASM228892v3
Denticeps clupeoides	denticle herring	299321	fDenClu1.2
Dicentrarchus labrax	European seabass	13489	dlabrax2021
Dipodomys ordii	Ord’s kangaroo rat	10020	Dord 2.0
Dromaius novaehollandiae	emu	8790	droNov1
Drosophila melanogaster	Fruit fly	7227	BDGP6.46
Echeneis naucrates	live sharksucker	173247	fEcheNa1.1
Echinops telfairi	small Madagascar hedgehog	9371	TENREC
Electrophorus electricus	electric eel	8005	fEleEle1.pri
Eptatretus burgeri	Inshore hagfish	7764	Eburgeri 3.2
Equus asinus	ass	9793	ASM1607732v2
Equus caballus	horse	9796	EquCab3.0
Erinaceus europaeus	western European hedgehog	9365	HEDGEHOG
Erpetoichthys calabaricus	reedfish	27687	fErpCal1.1
Erythrura gouldiae	Gouldian finch	44316	GouldianFinch
Esox lucius	northern pike	8010	fEsoLuc1.pri
Falco tinnunculus	common kestrel	100819	FalTin1.0
Felis catus	domestic cat	9685	Felis catus 9.0
Ficedula albicollis	Collared flycatcher	59894	FicAlb1.5
Fukomys damarensis	Damara mole rat	885580	DMR v1.0
Fundulus heteroclitus	mummichog	8078	Fundulus heteroclitus-3.0.2
Gadus morhua	Atlantic cod	8049	gadMor3.0
Gallus gallus	chicken	9031	bGalGal1.mat.broiler.GRCg7b
Gallus gallus gca000002315v5	chicken	9031	GRCg6a
Gallus gallus gca016700215v2	chicken	9031	bGalGal1.pat.whiteleghornlayer.GRCg7w
Gambusia affinis	western mosquitofish	33528	ASM309773v1
Gasterosteus aculeatus	three-spined stickleback	481459	GAculeatus UGA version5
Geospiza fortis	medium ground-finch	48883	GeoFor 1.0
Gopherus agassizii	Agassiz’s desert tortoise	38772	ASM289641v1
Gopherus evgoodei	Goodes thornscrub tortoise	1825980	rGopEvg1 v1.p
Gorilla gorilla	Western Lowland Gorilla	9595	gorGor4
Gouania willdenowi	blunt-snouted clingfish	441366	fGouWil2.1
Haplochromis burtoni	Burton’s mouthbrooder	8153	AstBur1.0
Heterocephalus glaber female	naked mole-rat	10181	Naked mole-rat maternal
Heterocephalus glaber male	naked mole-rat	10181	Naked mole-rat paternal
Hippocampus comes	tiger tail seahorse	109280	H comes QL1 v1
Homo sapiens	Human	9606	GRCh38
Hucho hucho	huchen	62062	ASM331708v1
Ictalurus punctatus	channel catfish	7998	ASM400665v3
Ictidomys tridecemlineatus	thirteen-lined ground squirrel	43179	SpeTri2.0
Jaculus jaculus	Lesser Egyptian jerboa	51337	JacJac1.0
Junco hyemalis	dark-eyed junco	40217	ASM382977v1
Kryptolebias marmoratus	mangrove rivulus	37003	ASM164957v1
Labrus bergylta	ballan wrasse	56723	BallGen V1
Larimichthys crocea	large yellow croaker	215358	L crocea 2.0
Lates calcarifer	barramundi perch	8187	ASB HGAPassembly v1
Laticauda laticaudata	blue-ringed sea krait	8630	latLat 1.0
Latimeria chalumnae	coelacanth	7897	LatCha1
Lepidothrix coronata	blue-crowned manakin	321398	Lepidothrix coronata-1.0
Lepisosteus oculatus	Spotted gar	7918	LepOcu1
Leptobrachium leishanense	Leishan spiny toad	445787	ASM966780v1
Lonchura striata domestica	Bengalese finch	299123	LonStrDom1
Loxodonta africana	African savanna elephant	9785	loxAfr3
Lynx canadensis	Canada lynx	61383	mLynCan4 v1.p
Macaca fascicularis	Crab-eating macaque	9541	Macaca fascicularis 6.0
Macaca mulatta	Macaque	9544	Mmul 10
Macaca nemestrina	Pig-tailed macaque	9545	Mnem 1.0
Malurus cyaneus samueli	superb fairywren	2593467	mCya 1.0
Manacus vitellinus	golden-collared manakin	328815	ASM171598v2
Mandrillus leucophaeus	Drill	9568	Mleu.le 1.0
Marmota marmota marmota	Alpine marmot	9994	marMar2.1
Mastacembelus armatus	zig-zag eel	205130	fMasArm1.2
Maylandia zebra	zebra mbuna	106582	M zebra UMD2a
Meleagris gallopavo	turkey	9103	Turkey 5.1
Melopsittacus undulatus	budgerigar	13146	bMelUnd1.mat.Z
Meriones unguiculatus	Mongolian gerbil	10047	MunDraft-v1.0
Mesocricetus auratus	Golden Hamster	10036	MesAur1.0
Microcebus murinus	gray mouse lemur	30608	Mmur 3.0
Microtus ochrogaster	vole	79684	MicOch1.0
Mola mola	ocean sunfish	94237	ASM169857v1
Monodelphis domestica	gray short-tailed opossum	13616	ASM229v1
Monodon monoceros	narwhal	40151	NGI Narwhal 1
Monopterus albus	swamp eel	43700	M albus 1.0
Moschus moschiferus	Siberian musk deer	68415	MosMos v2 BIUU UCD
Mus caroli	Ryukyu mouse	10089	CAROLI EIJ v1.1
Mus musculus	mouse	10090	GRCm39
Mus musculus 129s1svimj	mouse	10090	129S1 SvImJ v1
Mus musculus aj	mouse	10090	A J v1
Mus musculus akrj	mouse	10090	AKR J v1
Mus musculus balbcj	mouse	10090	BALB cJ v1
Mus musculus c3hhej	mouse	10090	C3H HeJ v1
Mus musculus c57bl6nj	mouse	10090	C57BL 6NJ v1
Mus musculus casteij	mouse	10091	CAST EiJ v1
Mus musculus cbaj	mouse	10090	CBA J v1
Mus musculus dba2j	mouse	10090	DBA 2J v1
Mus musculus fvbnj	mouse	10090	FVB NJ v1
Mus musculus lpj	mouse	10090	LP J v1
Mus musculus nodshiltj	mouse	10090	NOD ShiLtJ v1
Mus musculus nzohlltj	mouse	10090	NZO HlLtJ v1
Mus musculus pwkphj	mouse	39442	PWK PhJ v1
Mus musculus wsbeij	mouse	10092	WSB EiJ v1
Mus pahari	Shrew mouse	10093	PAHARI EIJ v1.1
Mus spicilegus	steppe mouse	10103	MUSP714
Mus spretus	algerian mouse	10096	SPRET EiJ v1
Mustela putorius furo	Domestic ferret	9669	MusPutFur1.0
Myotis lucifugus	little brown bat	59463	Myoluc2.0
Myripristis murdjan	pinecone soldierfish	586833	fMyrMur1.1
Naja naja	Indian cobra	35670	Nana v5
Nannospalax galili	Upper Galilee mountains blind mole rat	1026970	S.galili v1.0
Neogobius melanostomus	round goby	47308	RGoby Basel V2
Neolamprologus brichardi	lyretail cichlid	32507	NeoBri1.0
Neovison vison	American mink	452646	NNQGG.v01
Nomascus leucogenys	Northern white-cheeked gibbon	61853	Nleu 3.0
Notamacropus eugenii	tammar wallaby	9315	Meug 1.0
Notechis scutatus	mainland tiger snake	8663	TS10Xv2-PRI
Nothobranchius furzeri	turquoise killifish	105023	Nfu 20,140,520
Nothoprocta perdicaria	Chilean tinamou	30464	notPer1
Numida meleagris	helmeted guineafowl	8996	NumMel1.0
Ochotona princeps	American pika	9978	OchPri2.0-Ens
Octodon degus	Degu	10160	OctDeg1.0
Oncorhynchus kisutch	coho salmon	8019	Okis V2
Oncorhynchus mykiss	rainbow trout	8022	USDA OmykA 1.1
Oncorhynchus tshawytscha	Chinook salmon	74940	Otsh v2.0
Oreochromis aureus	blue tilapia	47969	ZZ aureus
Oreochromis niloticus	Nile tilapia	8128	O niloticus UMD NMBU
Ornithorhynchus anatinus	platypus	9258	mOrnAna1.p.v1
Oryctolagus cuniculus	rabbit	9986	OryCun2.0
Oryzias javanicus	javanese ricefish	123683	OJAV 1.1
Oryzias latipes	Japanese medaka HdrR	8090	ASM223467v1
Oryzias latipes hni	Japanese medaka HNI	8090	ASM223471v1
Oryzias latipes hsok	Japanese medaka HSOK	8090	ASM223469v1
Oryzias melastigma	Indian medaka	30732	Om v0.7.RACA
Oryzias sinensis	Chinese medaka	183150	ASM858656v1
Otolemur garnettii	small-eared galago	30611	OtoGar3
Otus sunia	Oriental scops-owl	257818	OtuSun1.0
Ovis aries	sheep	9940	ARS-UI Ramb v2.0
Ovis aries rambouillet	sheep	9940	ARS-UI Ramb v2.0
Pan paniscus	bonobo	9597	panpan1.1
Pan troglodytes	chimpanzee	9598	Pan tro 3.0
Panthera leo	lion	9689	PanLeo1.0
Panthera pardus	leopard	9691	PanPar1.0
Panthera tigris altaica	Tiger	74533	PanTig1.0
Papio anubis	olive baboon	9555	Panubis1.0
Parambassis ranga	Indian glassy fish	210632	fParRan2.2
Paramormyrops kingsleyae	Paramormyrops kingsleyae	1676925	PKINGS 0.1
Parus major	Great Tit	9157	Parus major1.1
Pavo cristatus	Indian peafowl	9049	AIIM Pcri 1.0
Pelodiscus sinensis	Chinese softshell turtle	13735	PelSin 1.0
Pelusios castaneus	West African mud turtle	367368	Pelusios castaneus-1.0
Periophthalmus magnuspinnatus	Periophthalmus magnuspinnatus	409849	PM.fa
Peromyscus maniculatus bairdii	Northern American deer mouse	230844	HU Pman 2.1
Petromyzon marinus	sea lamprey	7757	Pmarinus 7.0
Phascolarctos cinereus	koala	38626	phaCin unsw v4.1
Phasianus colchicus	Ring-necked pheasant	9054	ASM414374v1
Phocoena sinus	vaquita	42100	mPhoSin1.pri
Physeter catodon	sperm whale	9755	ASM283717v2
Piliocolobus tephrosceles	Ugandan red Colobus	591936	ASM277652v2
Podarcis muralis	common wall lizard	64176	PodMur 1.0
Poecilia formosa	Amazon molly	48698	PoeFor 5.1.2
Poecilia latipinna	sailfin molly	48699	P latipinna-1.0
Poecilia mexicana	shortfin molly	48701	P mexicana-1.0
Poecilia reticulata	guppy	8081	Guppy female 1.0 MT
Pogona vitticeps	central bearded dragon	103695	pvi1.1
Pongo abelii	Sumatran orangutan	9601	Susie PABv2
Procavia capensis	cape rock hyrax	9813	proCap1
Prolemur simus	greater bamboo lemur	1328070	Prosim 1.0
Propithecus coquereli	Coquerel’s sifaka	379532	Pcoq 1.0
Pseudonaja textilis	eastern brown snake	8673	EBS10Xv2-PRI
Pteropus vampyrus	large flying fox	132908	pteVam1
Pundamilia nyererei	Makobe Island cichlid	303518	PunNye1.0
Pygocentrus nattereri	red-bellied piranha	42514	fPygNat1.pri
Rattus norvegicus	Norway rat	10116	mRatBN7.2
Rattus norvegicus shrspbbbutx	Norway rat	10116	UTH Rnor SHRSP BbbUtx 1.0
Rattus norvegicus shrutx	Norway rat	10116	UTH Rnor SHR Utx
Rattus norvegicus wkybbb	Norway rat	10116	UTH Rnor WKY Bbb 1.0
Rhinolophus ferrumequinum	greater horseshoe bat	59479	mRhiFer1 v1.p
Rhinopithecus bieti	Black snub-nosed monkey	61621	ASM169854v1
Rhinopithecus roxellana	Golden snub-nosed monkey	61622	Rrox v1
Saccharomyces cerevisiae	baker’s yeast	559292	R64–1–1
Saimiri boliviensis boliviensis	Bolivian squirrel monkey	39432	SaiBol1.0
Salarias fasciatus	jewelled blenny	181472	fSalaFa1.1
Salmo salar	Atlantic salmon	8030	Ssal v3.1
Salmo trutta	brown trout	8032	fSalTru1.1
Salvator merianae	Argentine black and white tegu	96440	HLtupMer3
Sander lucioperca	pike-perch	283035	SLUC FBN 1
Sarcophilus harrisii	Tasmanian devil	9305	mSarHar1.11
Sciurus vulgaris	Eurasian red squirrel	55149	mSciVul1.1
Scleropages formosus	Asian bonytongue	113540	fSclFor1.1
Scophthalmus maximus	turbot	52904	ASM1334776v1
Serinus canaria	common canary	9135	SCA1
Seriola dumerili	greater amberjack	41447	Sdu 1.0
Seriola lalandi dorsalis	yellowtail amberjack	1841481	Sedor1
Sinocyclocheilus anshuiensis	blind barbel	1608454	SAMN03320099.WGS v1.1
Sinocyclocheilus grahami	golden-line barbel	75366	SAMN03320097.WGS v1.1
Sinocyclocheilus rhinocerous	horned golden-line barbel	307959	SAMN03320098 v1.1
Sorex araneus	European shrew	42254	COMMON SHREW1
Sparus aurata	gilthead seabream	8175	fSpaAur1.1
Spermophilus dauricus	Daurian ground squirrel	99837	ASM240643v1
Sphaeramia orbicularis	orbiculate cardinalfish	375764	fSphaOr1.1
Sphenodon punctatus	tuatara	8508	ASM311381v1
Stachyris ruficeps	rufous-capped babbler	181631	ASM869450v1
Stegastes partitus	bicolor damselfish	144197	Stegastes partitus-1.0.2
Strigops habroptila	Kakapo	2489341	bStrHab1 v1.p
Strix occidentalis caurina	northern spotted owl	311401	Soccid v01
Struthio camelus australis	African ostrich	441894	ASM69896v1
Suricata suricatta	meerkat	37032	meerkat 22Aug2017 6uvM2 HiC
Sus scrofa	pig	9823	Sscrofa11.1
Sus scrofa bamei	pig	9823	Bamei pig v1
Sus scrofa berkshire	pig	9823	Berkshire pig v1
Sus scrofa hampshire	pig	9823	Hampshire pig v1
Sus scrofa jinhua	pig	9823	Jinhua pig v1
Sus scrofa landrace	pig	9823	Landrace pig v1
Sus scrofa largewhite	pig	9823	Large White v1
Sus scrofa meishan	pig	9823	Meishan pig v1
Sus scrofa pietrain	pig	9823	Pietrain pig v1
Sus scrofa rongchang	pig	9823	Rongchang pig v1
Sus scrofa tibetan	pig	9823	Tibetan Pig v2
Sus scrofa usmarc	pig	9823	USMARCv1.0
Sus scrofa wuzhishan	pig	9823	minipig v1.0
Taeniopygia guttata	zebra finch	59729	bTaeGut1 v1.p
Takifugu rubripes	fugu	31033	fTakRub1.2
Terrapene carolina triunguis	Three-toed box turtle	2587831	T m triunguis-2.0
Tetraodon nigroviridis	spotted green pufferfish	99883	TETRAODON8
Theropithecus gelada	gelada	9565	Tgel 1.0
Tupaia belangeri	northern tree shrew	37347	TREESHREW
Tursiops truncatus	bottlenosed dolphin	9739	turTru1
Urocitellus parryii	Arctic ground squirrel	9999	ASM342692v1
Ursus americanus	American black bear	9643	ASM334442v1
Ursus maritimus	Polar bear	29073	UrsMar 1.0
Ursus thibetanus thibetanus	Asiatic black bear	441215	ASM966005v1
Varanus komodoensis	Komodo dragon	61221	ASM479886v1
Vicugna pacos	alpaca	30538	vicPac1
Vombatus ursinus	common wombat	29139	bare-nosed wombat genome assembly
Vulpes vulpes	red fox	9627	VulVul2.2
Xenopus tropicalis	tropical clawed frog	8364	UCB Xtro 10.0
Xiphophorus couchianus	Monterrey platyfish	32473	Xiphophorus couchianus-4.0.1
Xiphophorus maculatus	southern platyfish	8083	X maculatus-5.0-male
Zalophus californianus	california sea lion	9704	mZalCal1.pri
Zonotrichia albicollis	white-throated sparrow	44394	Zonotrichia albicollis-1.0.1
Zosterops lateralis melanops	silver-eye	1220523	ASM128173v1

## Methods

### Ensembl sequence retrieval

The Ensembl Representational State Transfer (REST) server application programming interface (API) [47] is used by TFBSFootprinter for automated retrieval of user-defined DNA sequences near the transcription start site of an established Ensembl transcript ID. Annotations for the transcript and Ensembl-defined regulatory regions (e.g., “promoter flanking region”) are also retrieved and mapped in the final output figure.

### PWMs

A total of 575 TF position frequency matrices (PFMs) retrieved from the JASPAR database [48] (http://jaspar.genereg.net/; nonredundant) are used to create PWMs (Eq. 1), as described by [49]:

(1) $$\mathrm{LL}S_{\mathrm{binding}}=\overset{N}{\underset{i=1}{\sum }}\log_{2}\left(\frac{\frac{a_{i}+\frac{b}{4}}{S+b}}{\frac{n_{\mathrm{nuc}}+b}{l_{\mathrm{bg}}+b}}\right)$$

N is the set of nucleotides in the currently scanned sequence; a_i_ is the number of instances of nucleotide *a* at position i; *b* is a pseudocount set to 0.8 per [49]; *S* is the number of sequences describing the motif; n_nuc_ is the count of the nucleotide in the background sequence; and l_bg_ is the length of the background sequence. The background frequencies for each nucleotide were set to match those of the human genome as determined previously [50].

### CAGE peak locations and Spearman correlation of expression values

Cap analysis of gene expression (CAGE) uses sequencing of cDNA generated from RNA to both determine TSSs and quantify their expression levels. The FANTOM project has performed CAGE across the human genome [46], and the results are freely available for download (http://fantom.gsc.riken.jp/data/). For non-human species “CAGE-like” peaks were derived using Ensembl RNA-Seq data for each of 218 species, as follows: 1) for each of 218 species, RNA-Seq data as BigWig files were downloaded (ensembl.org/pub/release-113/data_files/{species_name}), in most cases a single merged file was available but if not all available files were merged using Bedtools (version 2.31.1); 2) all subsequent peaks were filtered by width of >10 nt and signal strength >5 and merged if within 10 nt of one another; 3) genome annotations were downloaded as GTF files (ensembl.org/pub/release-113/gtf/{species_name}), and peaks occurring within 50 nt of an annotated TSS were associated to gene transcripts, and are described as “CAGE-like” peaks. Using the genomic locations of the FANTOM CAGE peaks or CAGE-like peaks (non-human species), the distances from each nucleotide position in the human genome to the nearest CAGE peak were calculated. The distribution of these distances was used to generate a log-likelihood score for all observed distances. The CAGE peak locations and distance/log-likelihood score pairings are then used during de novo prediction of TFBSs (Eq. 2).

(2) $$\mathrm{LL}S_{\mathrm{CAGE}\;\mathrm{distances}}=\overset{N}{\underset{i=1}{\sum }}-\log_{2}\left(P\left(x\leq d_{i}|\mathrm{genome}\right)\cdot \frac{p_{i}}{P_{\mathrm{total}}}\right)$$

Where N is the number of all CAGE peaks associated with the target gene; d_i_ is the distance to the current CAGE peak; p_i_ is the number of peak counts of the current CAGE peak; and p_total_ is the total peak count for this gene.

The expression data for CAGE peaks associated with the 575 JASPAR TF genes were then combined with the expression data for all CAGE peaks to perform a total of 386,652,770 Spearman correlation analyses via the “spearmanr” function from the SciPy Stats module [51]. Bonferroni correction was performed to account for multiple testing. Owing to the size of the analysis, a cutoff correlation magnitude value of 0.3 was used, and all the lower values (−0.3 <0.3) and correlation pairs were discarded. A distribution was generated from the resulting correlation data, which were used to generate log-likelihood scores for each possible correlation value (Eq. 3). The CAGE peak expression correlations/log-likelihood score pairings are then used during de novo prediction of TFBSs. Computation was performed via the supercomputing resources of the CSC – IT Center for Science Ltd.

(3) $$\mathrm{LL}S_{\mathrm{expression}\;\mathrm{correlation}}=-\log_{2}P\left(x\geq c_{\mathrm{current}}|c_{\mathrm{all}}\right)$$

Where c_current_ is the Spearman correlation between the expression of the target gene and the expression of the TF corresponding to the putative TFBS and where c_all_ is the distribution of all Spearman correlations between JASPAR TF genes and all genes.

### Experimental TFBSs compiled by the GTRD

The GTRD project (gtrd.biouml.org) is the largest comprehensive collection of uniformly processed human and mouse ChIP-Seq peaks and has compiled data from 8,828 experiments extracted from the Gene Expression Omnibus (GEO), Sequence Read Archive (SRA), and Encyclopedia of DNA Elements (ENCODE) databases [45]. One of the outputs of the performed analyses is reads that have been grouped to identify “metaclusters”, places where TF binding events cluster together in the human genome. We retrieved the metacluster data (28,524,954 peaks) from the GTRD database (version 18.0) [52] and subsequently mapped the number of overlapping metaclusters for each nucleotide position in the human genome. The distribution of these overlaps was used to generate a log-likelihood score for all observed overlap counts. The metacluster locations and distance/log-likelihood score pairings are then used during de novo prediction of TFBSs (Eq. 4).

(4) $$\mathrm{LL}S_{\mathrm{metaclusters}}=-\log_{2}P\left(x\geq n_{\mathrm{overlap}}|\mathrm{human}\;\mathrm{genome}\right)$$

Where n_overlap_ is the number of metaclusters overlapped by the current putative TFBS and where the D_human genome_ is the distribution of the number of overlapping metaclusters for every nucleotide position in the human genome.

### ATAC-Seq peaks

The assay for transposase-accessible chromatin using sequencing (ATAC-Seq) is an experimental method for revealing the location of open chromatin [53]. These locations are indicative of genomic regions that, owing to their unpacked nature, may allow TFs to bind to DNA and subsequently influence transcription. Open chromatin regions have been shown to be useful in the prediction of TFBSs [54]. We retrieved and compiled data from 135 ATAC-Seq experiments stored in the ENCODE project database (www.encodeproject.org) and mapped the distance from each nucleotide position in the human genome to the nearest ATAC-Seq peak, and the distribution of these distances was used to generate a log-likelihood score for all observed distances. The ATAC-Seq peak locations and distance/log-likelihood score pairings are then used during de novo prediction of TFBSs (Eq. 5).

(5) $$\mathrm{LL}S_{\mathrm{ATAC}-Seq\;\mathrm{distances}}=\overset{N}{\underset{i=0}{\sum }}-\log_{2}P\left(x\leq d_{i}|\mathrm{human}\;\mathrm{genome}\right)$$

Where N is the number of ATAC-Seq peaks within the current target region; d_i_ is the distance to the current ATAC-Seq peak; and D_human genome_ is the distribution of the distances to the nearest ATAC-Seq peak for each nucleotide position in the human genome.

### eQtls

The genome tissue expression (GTEX) project (gtexportal.org; version 7) has performed expression quantitative trait loci (eQTL) analysis on 10,294 samples from 48 tissues from 620 persons [36,55]. This analysis identified 7,621,511 variant locations in the genome, usually 1–5 base pairs (bp), that affect gene expression. eQTL data were extracted from the GTEX database and used to construct a distribution of the magnitude of effect on gene expression, which was then used to generate log-likelihood scores (Eq. 6). Next, we generated a second distribution of the distance from each gene to its variants; the distance was limited to 1,000,000 bp from either end of the transcript, as this is the search area over which GTEx scans for variants affecting the expression of each gene. The variant locations, magnitude of effect/log-likelihood score pairings, are then used during de novo prediction of TFBSs.

(6) $$\mathrm{LL}S_{\mathrm{eQTL}\;\mathrm{magnitude}}=\overset{N}{\underset{i=0}{\sum }}-\log_{2}P\left(x\geq m_{i}\;|\;\mathrm{human}\;\mathrm{genome}\right)$$

Where N is the number of eQTLs overlapping the current putative TFBS; m_i_ is the magnitude of effect of an eQTL overlapping the current putative TFBS; and D_human genome_ is the distribution of all eQTL magnitudes in each nucleotide position in the human genome.

### CpG islands

Because the methylation of DNA acts as a repressor of transcription, active promoters tend to be unmethylated. When methylated, the cytosine in a CpG dinucleotide can deaminate to thymine. Therefore, a CpG ratio close to what would be expected by chance is often indicative of an active promoter region [56,57]. Subsequently, CpG ratios (observed/expected) across a 200 nucleotide (nt) window were computed for each nucleotide position in the target genome. A distribution of these ratios was generated and used to generate log-likelihood scores for each possible ratio (Eq. 7). CpG ratio/log-likelihood score pairings are then used during de novo prediction of TFBSs.

(7) $$\mathrm{LL}S_{\mathrm{CpG}}=-\log_{2}P\left(x\geq r_{\mathrm{obs}/exp}\mathrm{target}\;\mathrm{genome}\right)$$

Where r_obs/exp_ is the ratio of observed to expected CpG dinucleotides in a 200 bp window centered on the current putative TFBS and where D_genome_ is the distribution of r_obs/exp_ across all nucleotide locations in the target genome.

### Conservation of vertebrate DNA

Conservation of sequence analysis has been performed by Ensembl to identify constrained elements for each species in each species group via the genomic evolutionary rate profiling (GERP) tool [58]. For each of the vertebrate species of Ensembl release 94, we calculated the distance from all nucleotides in the associated species genome to the nearest GERP constrained element and generated distributions of distances that were used to calculate log-likelihood scores for each distance (Eq. 8). GERP element distance/log-likelihood score pairings for each species are then used during de novo prediction of TFBSs in the relevant species.

(8) $$\mathrm{LL}S_{\mathrm{conservation}}=-\log_{2}P\left(x\leq d_{i}\;|\;\mathrm{target}\;\mathrm{genome}\right)$$

Where d_i_ is the distance between the current putative TFBS and the nearest conserved element in an alignment of 70 mammalian genomes (GERP) and D_genome_ is the distribution of distances between all nucleotides in the target genome and the nearest GERP conserved element.

### Combined affinity score

The addition of likelihood values is an established mathematical approach for measuring the combined effect of several independent parameters [59,60]. A summation of the weight (log-likelihood) scores from each experimental dataset is then performed for each putative TFBS and is represented as the “combined affinity score”. For analysis of human sequences, this is represented by Eq. 9. Owing to the limitations of available experimental data for nonhuman species, currently, for nonhuman vertebrates, the combined affinity score is described by Eq. 10. Complete scoring of ~ 80,000+ transcript promoter regions (1,000 bp) was used to generate *p* values for combined affinity scoring; computation was performed via the supercomputing resources of the CSC – IT Center for Science Ltd.

(9) $$\mathrm{Combined}\;\mathrm{Affinit}y_{\mathrm{human}}=Eq_{1}+Eq_{2}+Eq_{3}+Eq_{4}+Eq_{5}+Eq_{6}+Eq_{7}+Eq_{8}$$

(10) $$\mathrm{CombinedAffinit}y_{\mathrm{non}-\mathrm{human}}=Eq_{1}+Eq_{2}+Eq_{7}+Eq_{8}$$

### Benchmarking of several TFBS prediction tools

For comparison, in addition to TFBSFootprinter, several other TFBS prediction tools/models were used in benchmarking: the traditional PWM, DeepBind (v. 011; SELEX and ChIP-Seq models) [61], DeepSEA [62], and FIMO (meme v. 5.5.1; JASPAR 2018 nonredundant models) [63]. DeepBind was taken as an example utilizing a modern deep learning algorithm, and FIMO was chosen because it outperformed all other methods in a previous benchmark of de novo TFBS prediction tools [16]. For DeepBind, all parameters were set as defaults, analyses were performed with TF motifs on the basis of both SELEX and ChIP-Seq (when available), and the better of the two scores was retained. For DeepSEA, all parameters were set as defaults, analyses were performed with all 21,907 models, and then scores for all models matching the target TF were extracted and the best score kept. For TFBSFootprinter and FIMO, the *p* value threshold was set to 1, and all other settings were run as defaults. The TFBSFootprinter, FIMO, and PWM approaches all use JASPAR 2018 nonredundant TF motifs as the basis for scoring. For all the models, the top de novo prediction score for a target region (true positive or true negative) was kept as representative. For each TF, the correlating true positive and true negative scores were used to generate receiver operating characteristic (ROC) curves and quantify the area underneath (AUROC) via the “roc_curve” module of the scikit-learn Python library [64], which is a common method for evaluating TFBS prediction [61,65–67].

### Benchmarking on experimentally verified TFBSs

Experimentally verified and curated TFBSs belonging to the annotated regulatory binding sites (ABS) [68], ORegAnno [69], and Pleiades promoter project [70] databases were retrieved as GFF files from the Pazar database [71]. From these data, 504 experimentally validated binding sites affecting gene expression for 20 DeepBind TFs and 607 experimentally validated binding sites affecting gene expression for 25 JASPAR 2018 nonredundant TFs were selected. TFs were included only if they had at least 10 unique, experimentally validated TFBSs linked to gene expression changes, resulting in a final list of 14 TFs which could be compared across all prediction methods.

All target sites were converted from Hg19 to GRCh38 genomic coordinates via Ensembl REST. Subsequently, 50 bp sequences centered on each experimentally validated functional binding site in the human genome were retrieved to serve as true positives. The window length of 50 bp was chosen because it is wide enough to contain the longest TF motif (21 positions), which may overlap with the experimentally validated location at either end while also permitting some inaccuracy as to the exact center of the verified TFBS.

For each true positive, 50 true negatives were generated. True negatives were drawn at random locations within the promoter of the same Ensembl transcript of the corresponding true positive, within a 2,000 bp window (upstream and downstream) centered on each true positive, and at least 25 bp away.

### Analyzing the effect of multiomic transcription-relevant data on TFBS prediction

In addition to TFBSFootprinter benchmark scoring using all multiomic features, all 128 possible combinations of transcription-relevant features (PWM, CAGE, eQTL, metaclusters, ATAC-Seq, CpG, sequence conservation, expression correlation), which include PWM as one of the components, were used in scoring the true positives and true negatives. This allowed the identification of the best possible feature-combination TFBSFootprinter model for each TF, labeled “TFBSFootprinter best by TF”, as well as the TFBSFootprinter model, which performed best on average across all TFs, labeled “TFBSFootprinter best overall”. In the assessment of the DeepBind tool, both available models, which are based on SELEX or ChIP-Seq data, were used. Using a paired-sample t test, comparisons of ROC scores were made between all of the models: TFBSFootprinter, PWM, DeepBind, DeepSEA, and FIMO.

### Benchmarking on non-human species – mouse and zebrafish

Unlike in humans, extensive experimentally verified and curated TFBSs are not commonly available for the great majority of the 316 non-human species which the TFBSFootprinter tool can be used to analyze. As a result, benchmarking was performed using ChIP-Seq peaks for representative model organisms of mouse (Mus musculus) and zebrafish (Danio rerio). ChIP-Seq data for 225 TFs was retrieved from the ChIP-Atlas [72] database for mouse (genome assembly GRCm38/mm10). These GRCm38 genome assembly peak coordinates were translated to GRCm39 genome assembly coordinates using Liftover [73]. ChIP-Seq data for 10 TFs was retrieved from the GTRD database [45] for zebrafish (genome assembly GRCz11).For both mouse and zebrafish analyses, for each TF we mapped each of up to the top 500 ChIP-Seq peaks (a minimum threshold of 100 peaks was set) for that TF to their nearest transcript in the target genome and used the TFBSFootprinter tool to scan a region of 200 bp centered on the center of the ChIP-Seq peak (ChIP-Seq peaks ranged in width from several 10s to 100s of base pairs). These results served as true positives. For each true positive we derived a matching true negative in the following manner: take the same TSS relative coordinates of the true positive (total of 200 bp window) but apply them to another random transcript ID in the genome of the target species which has not had a ChIP-Seq peak for the current TF matched to it, and likewise analyze this region with TFBSFootprinter. In this way the 200 bp region analyzed is the same distance from the TSS and thus, presumably, is equivalent in potential binding/functionality. For each TF, the correlating true positive and true negative scores were used to generate ROC curves and quantify the AUROC via the “roc_curve” module of the scikit-learn Python library [64].

## Results

### Experimental datasets used in TFBS identification

**Figure 1.**
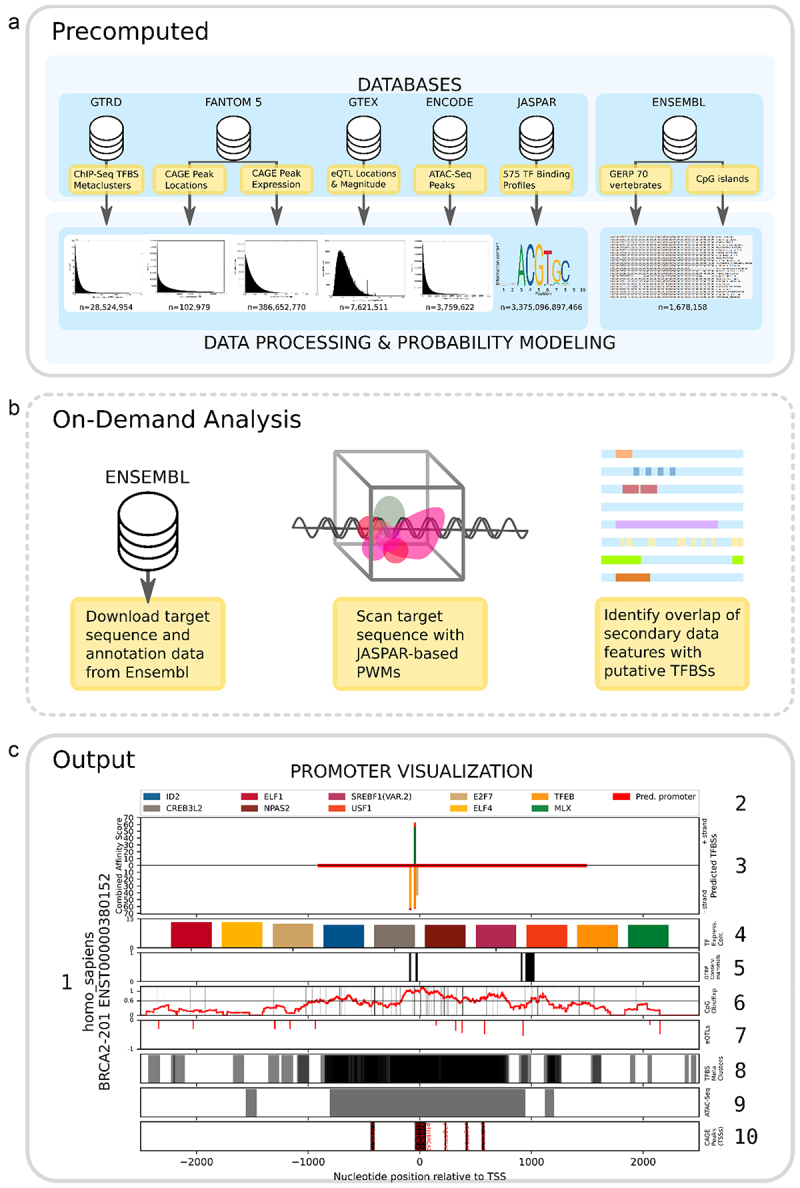
Outline of the datasets used in TFBSFootprinter. (a) A total of six empirical datasets are used to support the computational prediction of TFBSs via the TFBSFootprinter tool. The experimental data have been preprocessed to generate score distributions from which probability scores can be applied to putative TFBSs (*n* values indicate the number of elements used to compute distributions). (b) The user defines a target ensembl transcript ID and TSS-related start/end sites, which are then used to download the corresponding DNA sequence and annotation data via the ensembl API. PWM analysis of the DNA sequence generates putative TFBS hits, which are then compared with elements from the experimental datasets and scored via pregenerated log-likelihood scores relevant for the target genome. (c) The outputs of the TFBSFootprinter analysis are a table of results, including predicted TFBS names, locations, and scoring for each metric (not pictured), as well as individual files containing sequences and annotations. A publication-ready scalable vector graphics file (.Svg) is also produced, containing several elements as indicated and described. (C1) HUGO gene nomenclature committee (HGNC)-based identifier + ensembl transcript ID. (C2) color-coded legend of the top 10 TFs predicted to bind to this promoter. (C3) graphical representation of the promoter of the transcript where the predicted binding sites are indicated by colored bars. The bar height indicates the combined affinity score, and the bars on the positive y-axis indicate binding on the positive (sense) strand, and the negative y-axis represents the negative (antisense) strand. (C4) log-likelihood score of the correlation of expression between each top predicted TF gene and the target gene. (C5) highly conserved regions of 70-mammal alignment as determined by GERP analysis (black bars). (C6) vertical lines represent CpG locations. The red line indicates the CpG ratio of the promoter sequence over a 200 bp window. (C7) genetic variants identified in the GTEx database that affect target gene expression (eQtls). Green indicates a positive impact on expression (positive y-axis), and red indicates a negative impact (negative y-axis). (C8) TFBS metaclusters identified in the GTRD database (gray bars). (C9) ATAC-seq peaks (open chromatin) across many different cell types retrieved from the ENCODE database (gray bars). (C10) CAGE peaks indicating TSSs identified in the FANTOM database (black bars). The nucleotide positions at the bottom are relative to the ensembl-defined transcription start site of the target transcript and apply to C3 and C5–C10.

An outline of the TFBSFootprinter methodology is given in Figure 1, including the results of an example analysis of the DNA damage repair gene BRCA2 (BReast CAncer gene 2) Ensembl transcript ENST00000380152. Importantly, the results of this example prediction match previously experimentally verified TFBSs in the BRCA2 promoter, specifically sites for USF1, ELF1, and E2F family factors [74]. Several of the other predictions are for proteins that have a known role in both DNA damage, NPAS2 [75] and ID2 [76], and breast cancer [76,77].

Experimental data from a total of six databases were incorporated into the TFBSFootprinter algorithm for analysis of human genes (Figure 1(a)). Data from the relevant datasets were preprocessed to generate score distributions with which putative TFBS predictions could later be compared, as described in the Methods. Each dataset allows for scoring of transcription-relevant markers in or near putative regulatory elements identified by PWM analysis: colocalization with ChIP-Seq metaclusters; cap analysis of gene expression (CAGE) peaks, ATAC-Seq peaks, or CpG islands; correlation of expression between predicted TFs and genes of interest; colocalization of eQTLs and effects on the expression of target genes; and measurement of conservation in related vertebrate species (Figure 1(c)). For nonhuman vertebrates, analyses are performed on the basis of preprocessed data for PWM, CpG, and conservation. The simplicity of this piecewise approach allows for easy inclusion of additional TFBS-relevant data in the future.

### TFBSFootprinter availability

The TFBSFootprinter tool (https://github.com/thirtysix/TFBS_footprinting3) is available for installation via Conda (https://anaconda.org/thirtysix/tfbs-footprinting3) and as a Python library (https://pypi.org/project/TFBS-footprinting3/) and can subsequently be easily installed on a Linux system via the single command “pip install TFBS-footprinting3”. Owing to size considerations, supporting experimental data for both human and nonhuman species are downloaded on demand on first usage. Documentation on background, usage, and options is available both within the program and more extensively online (tfbs-footprinting.readthedocs.io). A listing and description of the analysis command line parameters are given in Table 2.

**Table 2.** TFBSFootprinter parameters.

Parameter	Values	Description
–t_ids_file, -t	[Full path to filename]	Required for running an analysis. Location of a file containing Ensembl target_species transcript ids. Input options are either a text file of Ensembl transcript ids or a.csv file with individual values set for each parameter.
–tf_ids_file, -tfs	[Full path to filename]	Optional: Location of a file containing a limited list of Jaspar TFs to use in scoring DNA sequence [default: all Jaspar TFs].
–promoter_before_tss, -pb	0–100,000; default, 900	Number (integer) of nucleotides upstream of TSS to include in analysis. If this number is negative the start point will be downstream of the TSS, the end point will then need to be further downstream.
–promoter_after_tss, -pa	0–100,000; default, 100	Number (integer) of nucleotides downstream of TSS to include in analysis. If this number is negative the end point will be upstream of the TSS. The start point will then need to be further upstream.
–top_x_tfs, -tx	1–20; default, 10	Number (integer) of unique TFs to include in output.svg figure.
–pval, -p	0.0000001–1; default 0.01	P value (float) for PWM score cutoff.
–pvalc, -pc	0.0000001–1; default 0.01	P value (float) for combined affinity score score cutoff.
-exp_data_update, -update		Download the latest experimental data files for use in analysis. Will run automatically if the “data” directory does not already exist (e.g., first usage).
–nofig, -no		Do not output a figure.

### The inclusion of empirical datasets improves TFBS prediction accuracy

**Figure 2.**
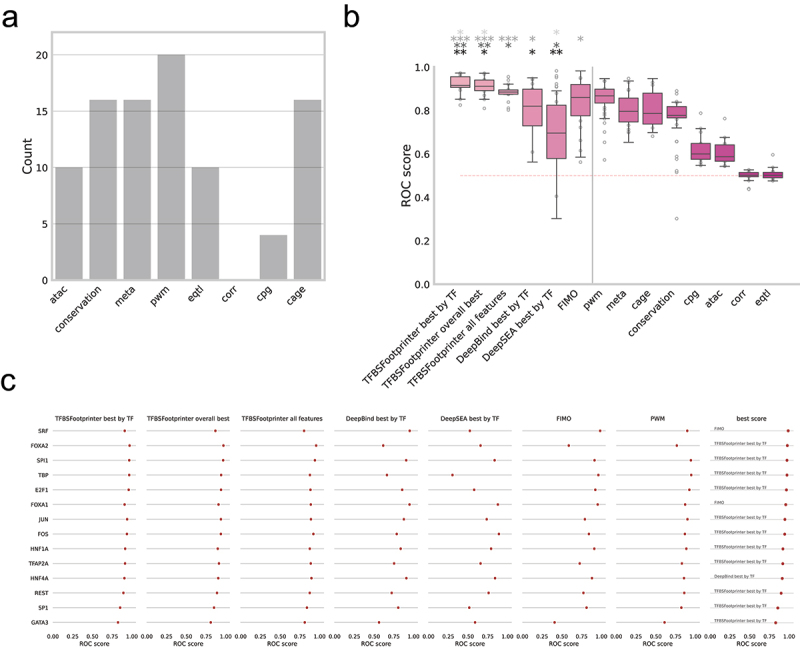
ROC analysis model performance in the identification of experimentally verified functional TFBSs—random locations in the same Ensembl transcript. ROC analysis was performed via experimentally verified functional TFBSs as annotated in the ORegAnno/Pleiades/ABS datasets as true positives, where true negatives were random locations in other Ensembl transcripts at the same distance from the TSS as the associated true positive. All the ROC curve analyses were performed on the TFs that had at least 10 true positives and at least 50 true negatives per true positive were used for each analysis. Each true positive/negative segment analyzed was 50 nucleotides long, and the highest TFBS score for the relevant dataset(s) was used for each true positive/negative segment. (a) bar plot of the frequency of experimental data types in the top 20 performing TFBSFootprinter models. (b) boxplot of ROC scores for TFBSFootprinter, DeepBind, DeepSEA, and FIMO for 14 TFs (left of vertical bar). ROC scores were also calculated using individual experimental metrics to show how well each contributes to accuracy of the combined model (right of vertical bar). (c) ROC scores for each individual TF tested for each primary TFBS prediction model under study. The best scoring model among all the models is named for each TF (right). TFBSFootprinter best by TF, which is based on using the highest ROC score achieved by some combination of experimental data models; TFBSFootprinter overall best, based on using the combination of experimental data models that had the best average ROC score across all the TFs analyzed; DeepBind best by TF, which is based on using the higher ROC score of the SELEX or ChIP-Seq DeepBind models. Black asterisks (bottom row) denote significant difference of first six models with ‘pwm’ model; dark gray asterisks (second from bottom row) denote significant difference between first six models and ‘DeepBind’ model; light gray asterisks (second from top row)denote significant difference between first six models and ‘DeepSEA’ model; silver asterisks (top row) denote significant difference between first six models and ‘FIMO’ model; as determined by related t-test.

The performance of both individual datasets and combinations of datasets in the identification of experimentally verified functional TFBSs was tested via ROC analysis (Figure 2, Table 3). Across the 14 TFs tested with all methods, the average AUROC were TFBSFootprinter with all features (0.881), TFBSFootprinter overall best (0.910), TFBSFootprinter best by TF (0.919); for the other models the values were DeepBind best by TF (0.798), DeepSEA best by TF (0.682), FIMO (0.817), and PWM (0.854). The ROC curves for TFBSFootprinter overall best and best by TF are included as Supplementary Figure S1.

*p-value < 0.05, **p-value < 0.005, and ***p-value < 0.0005

**Table 3.** AUROC results of the TFBS prediction method.

TF	TFBSFootprinter best by TF	TFBSFootprinter overall best	TFBSFootprinter all features	DeepBind best by TF	DeepSEA	FIMO	PWM
SRF	0.910	0.869	0.802	0.950	0.522	0.982	0.896
FOXA2	0.972	0.971	0.955	0.615	0.657	0.585	0.763
SPI1	0.967	0.967	0.939	0.905	0.835	0.911	0.941
TBP	0.967	0.942	0.876	0.662	0.302	0.959	0.946
E2F1	0.960	0.940	0.883	0.854	0.576	0.922	0.923
FOXA1	0.908	0.908	0.887	0.948	0.877	0.952	0.869
JUN	0.941	0.939	0.889	0.876	0.735	0.789	0.899
FOS	0.938	0.938	0.921	0.785	0.890	0.840	0.865
HNF1A	0.916	0.899	0.875	0.835	0.791	0.908	0.883
TFAP2A	0.914	0.914	0.887	0.752	0.657	0.724	0.828
HNF4A	0.905	0.904	0.896	0.907	0.841	0.879	0.854
REST	0.894	0.889	0.874	0.722	0.759	0.771	0.857
SP1	0.852	0.851	0.838	0.804	0.515	0.810	0.821
GATA3	0.825	0.810	0.810	0.562	0.588	0.405	0.608

The TFBSFootprinter model with the best average area under the ROC curve (AUROC) across all the tested TFs was the combination of PWM, ATAC, CAGE, conservation, and metacluster data (TFBSFootprinter overall best); with an average AUROC of 0.910. A paired-sample t test revealed that this model was significantly better than PWM (*p* value, 9.28 × 10^−3^), DeepBind (*p* value = 4.91 × 10^−3^), DeepSEA (*p* value, 1.79 × 10^−4^), and FIMO (*p* value, 3.75 × 10^−2^) (Figure 2(b)). When all the transcription-relevant features were used, TFBSFootprinter outperformed DeepBind (*p* value = 3.29 × 10^−2^).

### TFBSFootprinter produces strong predictions in mouse and zebrafish

ROC analysis of ChIP-Seq peaks was performed with TFBSFootprinter for 225 TFs in mouse and 10 TFs in zebrafish. In mouse, we observed a mean ROC AUC score of 0.777, with the lowest scoring 6 TFs (PROX1, SOX11, FOXC2, SOX4, PAX7, and TCF4) falling in a range of 0.451 to 0.498 and thus considered uninformative in prediction. A further 12 fell in the range of 0.506 to 0.598, and the remaining 207 scored a ROC AUC greater than 0.601, the full results are presented as Supplementary Table 1. Significantly fewer ChIP-Seq experiments were available for zebrafish, with the 10 TFs scoring ROC AUC values ranging from 0.506 (FOXD3) to 0.950 (CTCF), and an overall mean value of 0.715, the full results are presented as Supplementary Table 2.

## Discussion

We have tested the newest version [4,3,5,6] of our method for the prediction of TFBSs and introduce a tool that allows analyses of promoters in 317 vertebrate species, with automatic sequence retrieval and analysis on the basis of the Ensembl transcript ID. The method leverages transcription-relevant data to augment the prediction of functional TFBSs beyond the classical PWM. In benchmarking, the TFBSFootprinter method scored evenly or better than the traditional PWM, DeepBind, DeepSEA, and FIMO models when all the transcription-relevant data were used in its scoring. Surprisingly, benchmarking revealed that several types of transcription-relevant data, specifically eQTLs and gene expression correlations between putative TFs and target genes, did not contribute significantly to the prediction of TFBSs. As a result, the combination of features that produced the highest average ROC score across all tested TFs was PWM, ATAC, CAGE, conservation, and metaclusters. In paired-sample t test analysis, the TFBSFootprinter model performs significantly better than all the other models do. In addition, we identified specific combinations of transcription-relevant data that produced the best ROC scores for each tested TF; these combinations may lead to customizing TF models in the future. The low performance of the features related to RNA expression may be the result of the use of data that are not tissue specific; this is grounds for further research, as TFs, like many other proteins, can have tissue-specific expression patterns.

The results of testing TFBSFootprinter on ChIP-Seq peaks in mouse and zebrafish were promising. In mouse we observed that 207 of 225 tested TFs had ROC AUC scores greater than 0.60, and likewise in zebrafish the result was 8 of 10 tested TFs. As with experimentally validated TFBSs, ChIP-Seq data itself is at best sparse outside of the most researched species, and completely absent in the vast majority of others. The GEO database contains ChIP-Seq experiments with BED file data for just 16 vertebrate species, which quickly tapers off in experiment count: after 18,771 experiments for Homo sapiens and 6,864 for Mus musculus there is a more than 100-fold drop-off for the next most common species, Rattus norvegicus with 66 experiments. While we may only infer that TFBSFootprinter prediction of functional TFBSs matches the performance we observed in humans, as it incorporates four of the five top-performing transcription-relevant data (Figure 2), we do have direct evidence from our analyses in mouse and zebrafish that ChIP-Seq peaks scores are well reproduced by the tool.

We believe that TFBSFootprinter provides an excellent way to predict TFBSs as a high-performing model overall but also because of the ease of use in working with many vertebrate organisms. TFBSFootprinter may therefore supplement current investigations into gene function or provide a means to perform larger-scale analyses of groups of related target genes. After analysis of a target transcript, a publication-ready figure depicting the top scoring TFBS candidates is produced. Additionally, a number of tables (.csv) and JavaScript Object Notation (.json) files presenting various aspects of the results are output. Primary among these is a list of computational predictions in the target species that are supported by empirical data, which are sorted by a sum of the combined log likelihood scores (the combined affinity score). Importantly, scoring of nonhuman species is limited by the availability of external data for that species; at this time, the only data commonly available for nonhuman species are proximity to transcription hotspots (CAGE-like peaks), sequence conservation, CpG, and JASPAR motif data. Updates of species which are available for analysis are ongoing.

### TFBSFootprinter availability

The TFBSFootprinter tool is available as a Python package and can be installed via PyPi or Conda. Any Ensembl transcript ID from any of 317 vertebrate species available in the Ensembl database can be used as input. Starting with a list of Ensembl transcript ids for a target species (e.g., Homo sapiens), TFBSFootprinter will download a user-defined region of DNA sequence from the Ensembl server. The sequence is then scored using up to 575 JASPAR TFBS profiles or a more limited set as defined by the user. Each putative TFBS is then additionally scored on the basis of transcription-relevant data, which may include, depending on the target species, proximity/overlap with the TSS, TFBS metaclusters, open chromatin, eQTLs that affect expression levels of the proximal gene, conservation of sequence, correlation of expression with the proximal (target) gene, and CpG content.

### Limitations

In the benchmark, true negatives were defined on the basis of random locations that did not overlap with experimentally verified true positives. However, there is no guarantee that these sites are indeed devoid of any binding/functionality for the TFs in question. This is a notable problem in the testing of TFBS prediction, with random sites being one of the best solutions, although it is imperfect [66]. Not all vertebrate TF binding models cataloged in the JASPAR database are applicable to every vertebrate species, as not all species possess the same genes. Users will be required to ensure that any predicted TF has an ortholog in any species in which they perform TFBS prediction.

Two of the benchmarks were scored on ChIP-Seq data from the GTRD database, and because GTRD metaclusters are part of the TFBSFootprinter scoring model, some bias is introduced. However, metaclusters are defined by merging all ChIP-Seq data (all TFs, therefore TF agnostic) across all peak-calling methods (four separate peak-calling methods), and benchmarking was performed on ChIP-Seq data from one peak-calling method for individual TFs. We chose to perform TFBSFootprinter analysis using all the available transcription-relevant features. In the future, we plan to expand the testing and assessment of empirical datasets and incorporate an option to use the combination of features that is proven best for each individual TF.

TFBSFootprinter integrates multiple transcription-relevant features (e.g., conservation, ChIP-Seq metaclusters, CpG content, and TSS proximity) to support prediction of TFBSs; the tool is not intended for independent analysis of these features outside of their role in TFBS prediction.

## Data Availability

The TFBSFootprinter project page is located at https://github.com/thirtysix/TFBS_footprinting3. Results of benchmarking of TFBSFootprinter (https://osf.io/hzny6/) are available as Open Science Foundation repositories.

## Abbreviations

ATAC-Seq: Assay for transposase-accessible chromatin by sequencing
bp: Base pairs
CAGE): Cap analysis of gene expression
ChIP-Seq: Chromatin immunoprecipitation with massively parallel DNA sequencing
CRE): Cis-regulatory element
eQTL: Expression quantitative trait locus
HT-SELEX: High-throughput systematic evolution of ligands by exponential enrichment
PFM: Position frequency matrix
PWM: Position weight matrix
TF: Transcription factor
TFBS: Transcription factor binding site
TSS: Transcription start site
